# Influence of M-EMS on Fluid Flow and Initial Solidification in Slab Continuous Casting

**DOI:** 10.3390/ma14133681

**Published:** 2021-07-01

**Authors:** Guoliang Liu, Haibiao Lu, Bin Li, Chenxi Ji, Jiangshan Zhang, Qing Liu, Zuosheng Lei

**Affiliations:** 1 State Key Laboratory of Advanced Metallurgy, University of Science and Technology Beijing, Beijing 100083, China; liuguoliang1983@163.com (G.L.); zjsustb@163.com (J.Z.); 2 Shougang Group Co., Ltd., Research Institute of Technology, Beijing 100043, China; jicx5617@shougang.com.cn; 3 State Key Laboratory of Advanced Special Steel & Shanghai Key Laboratory of Advanced Ferrometallurgy, Shanghai University, Shanghai 200444, China; luhaibiao@shu.edu.cn (H.L.); gumufeng1023@i.shu.edu.cn (B.L.)

**Keywords:** continuous casting, electromagnetic stirring, flow behavior, uniform index of solidified shell

## Abstract

A mathematical model coupled with electromagnetic field has been developed to simulate the transient turbulence flow and initial solidification in a slab continuous casting mold under different electromagnetic stirring (EMS) currents and casting speeds. Through comparing the magnetic flux density, flow field with measured results, the reliability of the mathematical model is proved. The uniform index of solidified shell thickness has been introduced to judge the uniformity of the solidified shell. The results show that a horizonal recirculation flow has been generated when EMS is applied, and either accelerated or decelerated regions of flow field are formed in the liquid pool. Large EMS current and low casting speed may cause the plug flow near the mold narrow face and a suitable EMS current can benefit to the uniform growth of solidified shell. Meanwhile, an industrial test exhibits that EMS can weaken the level fluctuation and number density of inclusion. Overall, a rational EMS current range is gained, when the casting speed is 1.2 m/min, the rational EMS current is 500–600 A.

## 1. Introduction

Continuous casting has been widely developed as the most important production process in the steelmaking industry. During this process, molten steel flows into the mold through a submerged entry nozzle (SEN), solidifies against the water-cooled copper mold walls in the presence of many complex metallurgical phenomena, including multiphase flow, heat transfer, solidification and solute transport, and it finally forms a solidified shell [1]. Flow of molten steel in the mold and solidification in this process is of great importance because it is responsible for many surface and internal defects [2], such as hooks and longitudinal cracks. Therefore, it is important to control the flow and solidification within an acceptable process to avoid defects.

To enhance or even control the liquid steel flow, many techniques have been developed and used, including argon blowing [3], electromagnetic brake [4,5] (EMBr), electromagnetic stirring [6,7,8,9,10,11,12] (EMS), etc. Together with these techniques, EMS has been shown to be one of the most effective countermeasures to improve the molten steel flow and initial solidification in the mold and most works focused mainly on the rotary stirring in billet or round bloom.

Li et al. [9] developed a mathematical model coupling the electromagnetic and flow fields; it was developed to investigate the influence of the SEN clogging rate on the flow field and the influence of electromagnetic stirring (EMS) on the asymmetric mold flow. Fang et al. [13] and Ren et al. [14] simulated the flow, temperature and solidification field in billet mold, and the results showed that EMS can reduce the impinging effect of jet flow and eliminate molten steel superheat. Wang et al. [15] developed a 3-D coupled model considering electromagnetic field, flow field, heat transfer and level fluctuation under different EMS positions in continuous billet mold, and found that the velocity and wave height at steel/slag interface decreases under lower stirrer position. Maurya et al. [16,17,18] analyzed the influence of EMS on flow and solidified shell under different EMS currents and frequencies. However, little research considered the influence of EMS on the flow and initial solidification on slab.

In a series of works from Fujisaki [7,19,20] and his coworkers, a 3-D magnetohydrodynamic model was developed to evaluate the flow, heat transfer as well as free surface in mold with EMS separately, and the results showed that EMS makes the solidified shell uniform and the dynamic deviation of temperature stable. Li et al. [21] investigated the influence of EMS position on flow field, possibility of slag entrapment and inclusion removal in slab mold, the flow pattern in the mold changes greatly under different EMS positions and the low stirrer position is favorable to inclusion removal, but the solidified behavior of shells are not considered in his study. In addition, most of them mainly considered the influence of EMS current on the flow and heat transfer in the mold, while the influence of EMS under other process parameters, such as casting speed, SEN depth, etc. has not yet been investigated.

In this paper, the fluid flow and solidification in a continuous casting slab mold under EMS were numerically simulated by a multi-physics model. By comparison with the magnetic flux density data and flow field, the reliability of the mathematical model is proved. The uniform index of solidified shell thickness has been introduced to judge the uniformity or solidified shell. Afterwards, the metallurgical behaviors under different EMS currents and casting speeds were investigated and compared to obtain optimal process parameters with a relatively reasonable flow pattern and solidified shell. According to the simulation results, the industry test was carried out to estimate the influence of EMS on level fluctuation and number density of inclusion.

## 2. Mathematical Modeling

### 2.1. Assumption

In order to simplify the numerical simulation, the present work includes the following assumptions and simplifications:

(1) The influence of flow field on the electromagnetic field is ignored due to the small magnetic Reynolds number [22], and the electromagnetic field is assumed to be quasi-static.
(2) The influence of Joule heat generated by currents is ignored in simulation of heat transfer and solidification due to its low frequency.
(3) The liquid steel and the liquid slag behave as incompressible Newtonian fluids.
(4) The effects of mold oscillation and mold curvature are not taken into account [23].

### 2.2. Governing Equation

A transient three-dimensional mathematical model for slab continuous casting has been developed which was coupled with electromagnetic field, flow and heat transfer. The corresponding governing equations are written as follows:

#### 2.2.1. Electromagnetic Model

Electromagnetic field was determined by solving Maxwell’s equations:

(1) $$\nabla \cdot \vec{B}=0$$

(2) $$\nabla \times \vec{H}=\vec{J}$$

(3) $$\nabla \times \vec{E}=-\frac{\partial \vec{B}}{\partial t}$$

(4) $$\vec{J}=\sigma \left(\vec{E}\right)$$

where $\vec{B}$ is the magnetic flux density, σ is the electric conductivity, $\vec{E}$ is the electric field strength, V/m; $\vec{J}$ is the induced current density and $\vec{H}$ is the magnetic field strength.

The time-averaged electromagnetic force can be calculated by:

(5) F→=12Re(J→×B→*)

where $\vec{F}$ is the time-average electromagnetic volume force, ${\vec{B}}^{{}_{*}}$ is the complex conjugate of $\vec{B}$ and Re denotes the real part of the complex quantity.

#### 2.2.2. Fluid Flow and Solidification Model

Continuity equation:

(6) $$\nabla \cdot (\rho \vec{u})=0$$

Momentum equation (N-S):

(7) $$\frac{\partial (\rho \vec{u})}{\partial t}+\nabla \cdot (\rho \vec{u}\vec{u})=-\nabla p+\nabla \cdot (\mu_{eff}\nabla \vec{u})+\rho g+S_{m}+\vec{F}$$

where $\vec{u}$ is the fluid velocity, *p* is the pressure, *ρ* is the fluid density and *μ_eff_* is the effective viscosity, the standard *κ − ε* turbulent model is applied to calculate the effective viscosity, *μ_eff_*.

Energy equation,

(8) $$\frac{\partial }{\partial t}(\rho H)+\nabla \cdot (\rho \vec{u}H)=\nabla \cdot \left[(\lambda +C_{p}\frac{\mu_{t}}{{\mathrm{Pr}}_{t}})\nabla T\right]$$

where,

(9) $$H=h_{ref}+\int_{T_{ref}}^{T}c_{p}dT+f_{l}L_{s}$$

where *λ* is the thermal conductivity of the fluid, *C_p_* is the specific heat capacity of the fluid, *Pr_t_* is the turbulence Prandtl number (0.85), *H* is enthalpy, *h_ref_* is the reference enthalpy relative to temperature *T_ref_*, *f_l_* is liquid fraction at mushy zone and *L_s_* is latent heat of solidification.

The enthalpy-porous model is used to simulate the solidification of steel in a continuous slab casting mold, the liquid-solid mushy zone is treated as a porous zone. The sink (*S_m_*) is added to the momentum equations as a source term:

(10) $$S_{m}=\frac{{\left(1-f_{l}\right)}^{2}}{\left(f_{l}{}^{3}+x\right)}A_{mush}(\vec{u}-u_{c})$$

where, *A_mush_* is a mushy zone constant, 10^8^ [24] and *u_c_* is the casting speed.

### 2.3. Geometry Model and Boundary Conditions

#### 2.3.1. Geometry Model

**Figure 1:**
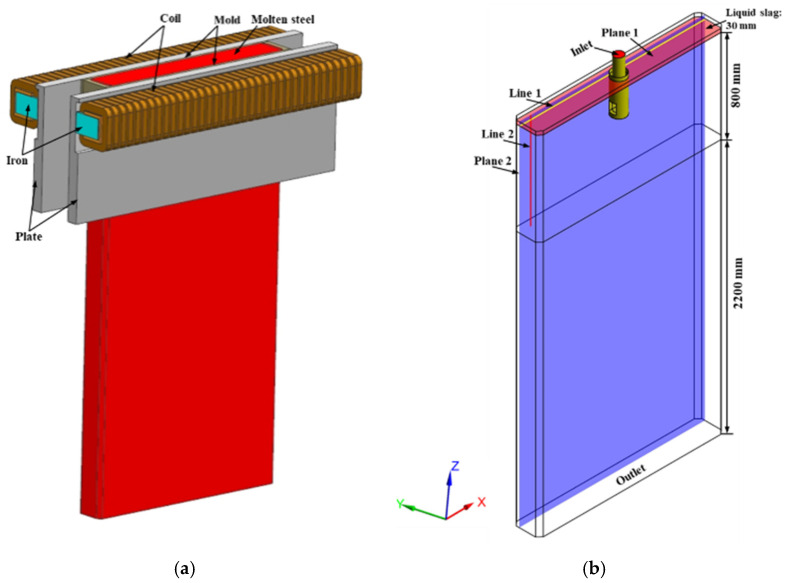
Schematics of the calculation model: (**a**) model used for electromagnetic simulation (surrounding air cuboid is not shown), (**b**) model used for fluid simulation.

A three-dimensional mathematical model was created that consists of submerged entry nozzle (SEN), Liu et al. [25] as the authors studied the chamfered slab mold in studied metals, a chamfered slab mold with the section of 1600 mm × 230 mm, length of 800 mm; considering the influence of lower recirculation flow on the flow field of molten steel in the mold, we extended the mold model to 3000 mm. Figure 1a shows the geometry model of an electromagnetic simulation, a pair of traveling-wave electromagnetic stirrers is designed on both sides of the mold wide face. The schematic of fluid simulation is presented in Figure 1b, and the other process parameters for numerical simulation are given in Table 1.

#### 2.3.2. Boundary Conditions for Electromagnetic Simulation

The whole geometry model of the electromagnetic field was taken to be surrounded by an air cuboid (2.95 m × 1.2 m × 3.6 m) in which most of the magnetic flux lines are closed. Boundary conditions are applied on the external surface of this cuboid with magnetically flux parallel boundary [14].

#### 2.3.3. Boundary Conditions for Fluid Field Simulation

(1) The inlet velocity of the SEN was calculated based on the mass conservation, and turbulent kinetic energy and the energy dissipation rate are estimated by the semi-empirical equations [26]. The casting temperature is set as 1827 K.
(2) The outlet boundary at the bottom of the calculation domain is a fully developed outflow condition.
(3) The mold wall is treated with the no-slip boundary condition and the heat flux on the wide and narrow faces is a function of distance toward the mold bottom, as shown in Equation (11), which is similar to the form proposed by Savage [27]. The convective heat transfer boundary condition is imposed on the extended region of the continuous caster, and the average heat transfer coefficient for wide and narrow faces is 320 W/(m^2^·K) and 360 W/(m^2^·K), respectively.
  (11) $$q=2.68-\varphi \sqrt{\frac{60L}{u_{c}}}$$
  where *q* is the heat flux density, *φ* represents constants for wide and narrow faces calculated according to the imported and exported temperature difference and the cooling water flow rate in the mold, which is 0.288 and 0.292, respectively, and *L* is the distance from the meniscus, m.
(4) The top surface is treated as a free-slipped boundary condition and considering the heat insulation of mold flux, adiabatic condition is applied to it.

### 2.4. Numerical Solution Procedure

In this study, the numerical simulation is divided into two sections: first, the magnetic field and Lorentz force are computed using the commercial software, ANSYS EMAG, by solving the Maxwell equations, and then the time-averaged electromagnetic force is interpolated into the momentum equation as a source term of ANSYS FLUENT. The calculation domain of fluid field model is divided into 2 million finite volumes and grid-independent verification was performed; the structured mesh is used to simulate the fluid flow and heat transfer in the slab mold. The pressure-implicit with splitting of operators (PISO) algorithm was used for the pressure-velocity coupling and first-order upwind is used for the discretization of momentum and energy equations and the time step size is 0.004 s. The physical parameters for numerical simulation are given in Table 2.

## 3. Results and Discussion

### 3.1. Validation of Electromagnetic Field and Flow Field

**Figure 2:**
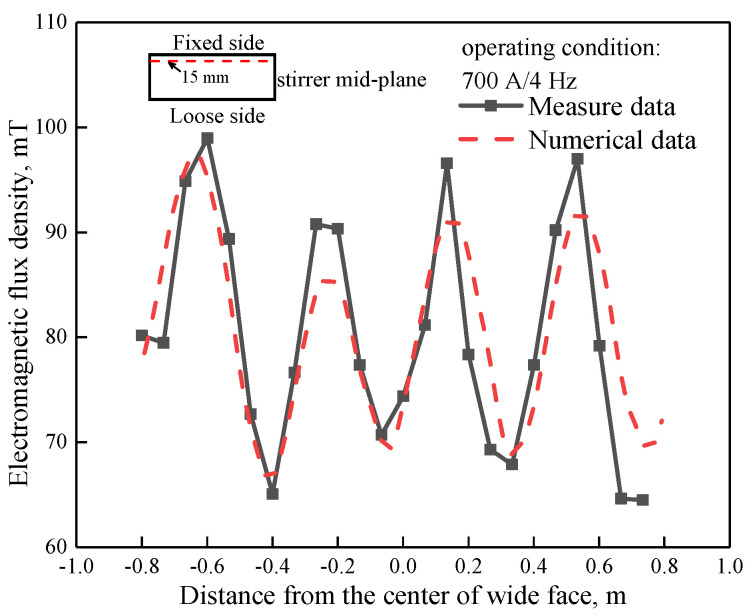
Distribution of measured and numerical magnetic flux density at stirrer mid-plane (Z = −0.075 m).

Figure 2 displays the distribution of measured and numerical magnetic flux density at 15 mm from the mold’s fixed side of the stirrer mid-plane with operating condition of 700 A/4 Hz, where magnetic flux density data was measured using a CT-3 Teslameter within an empty mold. It can be observed that the numerical results matched well with the measured data; both the predicted data and distribution tendency are similar, the biggest numerical magnetic flux density is about 98 mT, which was located near the left side of the mold.

**Figure 3:**
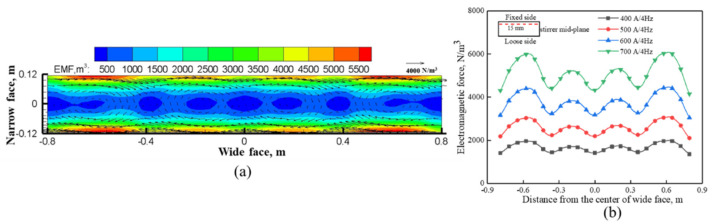
Distribution of (**a**) time-average electromagnetic force at the center of stirrer and (**b**) electromagnetic force under different EMS currents.

Figure 3a depicts the distribution of time-averaged electromagnetic force with the operating condition of 700 A/4 Hz, and it can be seen that the distribution of time-averaged electromagnetic force is centrosymmetric, which resulted from the similar distribution of magnetic flux density [9]. The tangential components of electromagnetic force are in the same direction at each edge, but their directions are opposite to each other, and thus, produce a horizontal recirculation. Four transverse swirls of electromagnetic force exist in the interior of the cross-section, which correspond to the pole number acting on the molten steel. Figure 3b illustrates the variation of electromagnetic force at 15 mm from the mold’s fixed side of the stirrer mid-plane under different EMS currents. It can be seen that electromagnetic force increases with the EMS current, as the EMS current increases from 400 to 800 A, the maximum electromagnetic force is 2133 N/m^3^ and 7950 N/m^3^.

In order to validate the mathematical model, a 1/5th scaled physical model using mercury when EMS is not applied was established; the fluid velocity in the mold was measured by means of ultrasound Doppler velocimetry (UDV), and Li et al. [30] as the authors published this apparatus’ description in *ISIJ International*. Meanwhile, a mathematical model was set corresponding to the physical model both on the geometry and material properties, etc.

**Figure 4:**
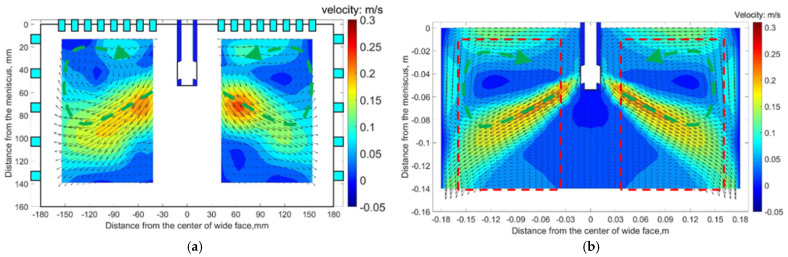
Vector and contour of fluid flow obtained at quarter plane of mold fixed side under the condition of 1.2 m/min-0 A: (**a**) physical model and (**b**) numerical model.

Figure 4a exhibits the flow pattern obtained from the physical model and Figure 4b exhibits the flow pattern obtained from the mathematical model at a quarter longitudinal plane near the fixed side of the mold. When EMS is not applied, a typical roll-flow pattern is observed, both the measured flow pattern and numerical flow pattern are similar not only in flow structure but also in characteristic points, for example, the impinging point at mold narrow face. It can be seen that the numerical results matched well with the experimental results. Overall, by comparison of the magnetic flux density and flow field between simulation and experimental results, the mathematical model is proven to be reliable.

### 3.2. Effect of EMS Current on Fluid Flow in the Mold

**Figure 5:**
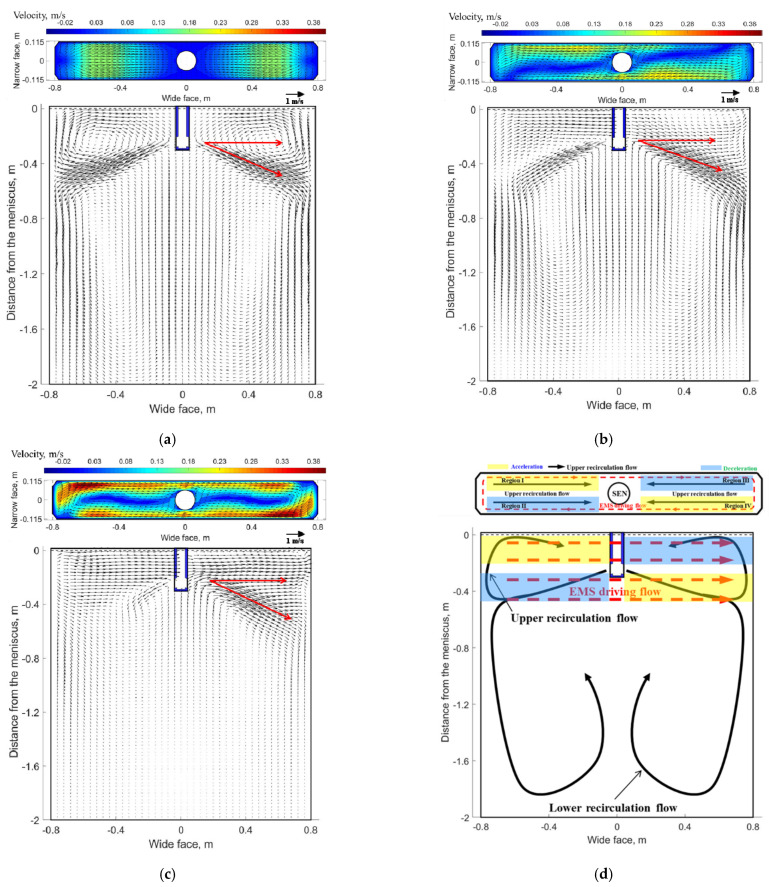
Velocity distribution at transverse plane (plane 1) and a quarter longitudinal plane (plane 2): (**a**) without-EMS, (**b**) 400 A-EMS, (**c**) 700 A-EMS (**d**) schematic of flow pattern with EMS.

In order to analyze the flow field inside the liquid pool, two planes and corresponding characteristic lines were selected, as indicated in Figure 1b. Plane 1 is a transverse plane which −5 mm distance from the meniscus, and line 1 is located at Y = 57.5 mm of plane 1, while plane 2 is a longitudinal plane at Y = 57.5 mm iso-surface, and line 2 is located at X = −700 mm of plane 2. The velocity distribution at plane 1 and plane 2 under different EMS currents when the casting speed is 1.2 m/min are shown in Figure 5.

As shown in Figure 5a, when EMS is off, the flow pattern at plane 2 is a classical double-roll structure as the jet travels to the narrow face and then splits into an upward (to the free surface and back towards the SEN) and downward (to carry molten steel deep into the mold) flow. For plane 1, molten steel impinges the free surface near the mold narrow face and results in a large velocity at this region, whereas the flow pattern near the SEN is almost stagnant. This nonuniform velocity distribution may affect the temperature distribution near the meniscus to some extent. EMS can improve this nonuniform distribution of velocity and temperature to some extent.

When EMS is applied (shown in Figure 5b,c), for plane 1, a horizonal recirculating flow has been generated due to the effect of electromagnetic force, this trend is similar to Yin’ work in [31]. Meanwhile, it can be seen that the velocity at both side of SEN increases significantly compared with that without EMS. For plane 2, the upper and lower recirculation flow disappears gradually, instead, molten steel at the left side tends to move towards the right side of mold. In fact, the flow pattern in the mold is the interaction of inertial flow caused by jet flow and driving flow induced by electromagnetic force; Figure 5d shows the schematic of flow pattern with EMS, two different accelerated and decelerated regions exist both at the longitudinal plane and transverse plane which are related to the direction of electromagnetic force and initial flow pattern without EMS. With the increase of EMS current, it can be seen that an integrated recirculation flow has been generated at plane 1, and the velocity magnitude also increases, while for plane 2, the jet flow has obviously been inhibited at the left side of the SEN, meanwhile, the jet angle at the right side of the SEN decreases due to large electromagnetic force.

**Figure 6:**
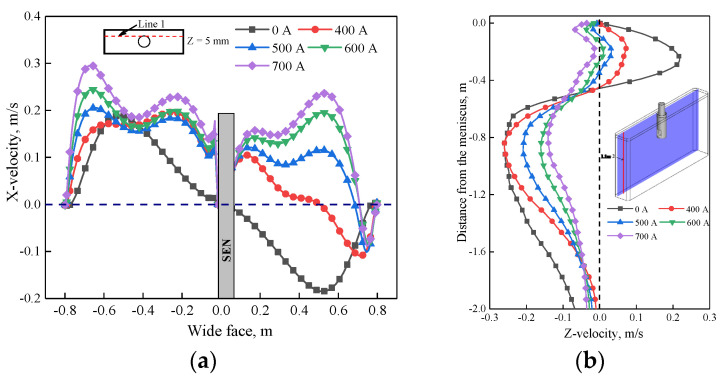
Variation of velocity under different EMS currents: (**a**) X-velocity at line 1, (**b**) Z-velocity at line 2.

Further justification of velocity under the influence of EMS is plotted in Figure 6. As shown in Figure 6a, when EMS is off, the X-velocity has the same magnitude at both sides of the SEN but with opposite direction; the maximum value is 0.18 m/s which corresponds to the impinging region of upper recirculation flow of Figure 5a. When EMS is on, with the increase of EMS current, the value of X-velocity at the left side of the SEN increases due to the accelerated region of Figure 5d, while for the right side of SEN, the direction of X-velocity changed firstly because of the horizonal swirling flow, and then the value of X-velocity increases with the increase of EMS current. For the small EMS current (400–600 A), it can be observed that the maximum value of X-velocity is less than that without EMS (0.18 m/s), it means that small parts of upper recirculation flow still exist which hinders the horizonal recirculation flow, while when EMS current is greater than 600 A, the maximum value of X-velocity is 0.21 m/s which is larger than 0.18 m/s, it indicates that the upper recirculation flow has been broken completely in Figure 6b, with the increase of EMS current, the velocity in the mold decreases, as the EMS current increases to 700 A, the Z-velocity all changes to negative, which indicates that a plug flow has generated near the mold narrow face. This flow pattern may not benefit to the removal of inclusion.

### 3.3. Effect of EMS Current on Initial Solidification

**Figure 7:**
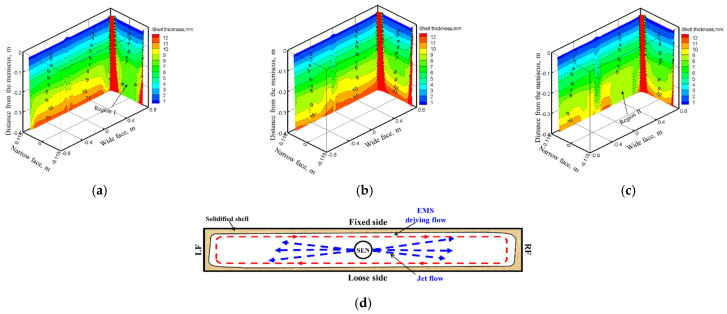
3D variation of solidified shell thickness along the casting direction under different EMS currents: (**a**) 0 A, (**b**) 400 A, (**c**) 700 A, (**d**) schematic of transverse flow near jet flow with EMS.

The 3D variation of solidified shell thickness along the casting direction under different EMS currents at the casting speed of 1.2 m/min are shown in Figure 7.

As shown in Figure 7, the initial solidified shell was generated at −0.042 m distance from the meniscus, and its thickness increases along the casting direction. When EMS is off, the jet flow pouring from the SEN impinges to the solidified shell at the narrow face of the mold, which may result in the thin and uneven distribution of solidified shell at the impact region. When EMS is applied, the solidified shell thickness at the mold narrow face increases because that the direct impact of jet flow has been weakened under the influence of electromagnetic force. While for the mold wide face, due to the washing effect of transverse flow, the uniformity of solidified shell thickness changes even for a suitable EMS current (Figure 7b), however, for the higher EMS current (700 A), the solidified shell thickness at the right side of wide face decreases The main reason is that as shown in Figure 7d, an electromagnetic force gradient exists from the edge to the center of the slab, the jet flow may deviate towards the mold wide face and lead to the remelting of solidified shell; this phenomenon may not benefit the uniformity of the solidified shell to any extent.

**Figure 8:**
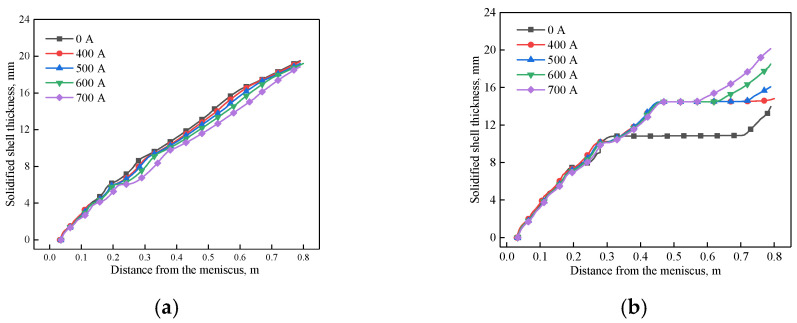
Variation of solidified shell at the center of: (**a**) wide face, (**b**) narrow face under different EMS currents.

Figure 8 shows the variation of solidified shell thickness at the center of the mold wide face and narrow face under different EMS currents, respectively. It can be seen that the solidified shell becomes thinner as the EMS current increases for the mold wide face. This phenomenon can be attributed to the superheat dissipation effect of EMS. While for the mold narrow face, when the EMS is not applied, it can be observed that the solidified shell stops growing due to the remelting phenomenon of high temperature liquid steel, which may increase the risk of break out. With the increase of EMS, the solidified shell thickness at the mold exit increases. When the EMS current increases from 0 to 700 A, the shell thickness of wide face and narrow face at the mold exit changes from 19.51 and 10.2 mm to 18.85 and 18.5 mm, respectively.

**Figure 9:**
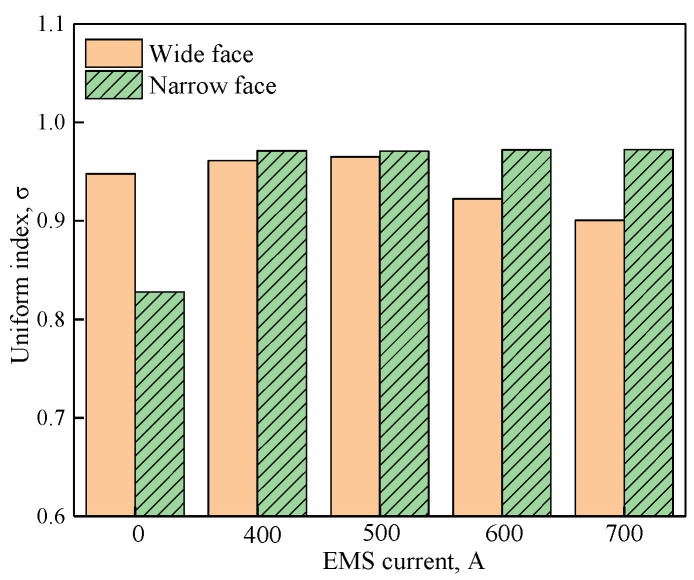
Uniform index at z = −0.4 m under different EMS currents.

In the continuous casting process, the initial solidified shell near the meniscus around the perimeter of the mold is of great importance because it is associated with crack formation [1]. Therefore, in order to describe the uniformity of solidified shell thickness quantitatively, the uniform index of solidified shell thickness is set up, *U*_i_, and the relevant definitions are depicted in Equations (12)–(14):

(12) $$U_{i}=1-\frac{\sigma_{i}}{h_{i-ave}}$$

(13) $$h_{i-ave}=\left(\sum_{j=1}^{m}h_{i-j}\right)/m_{i}$$

(14) $$\sigma_{i}=\sqrt{\frac{\sum_{j=1}^{m}{\left(h_{i-j}-h_{i-ave}\right)}^{2}}{m_{i}}}$$

where, *i* represents the wide face (WF) and narrow face (NF) at mold transverse plane, respectively. *h_i − j_*, *h_i − ave_* and *m_i_* are solidified shell thickness in mesh element *j*, averaged solidified shell thickness and total mesh element number, respectively, and *σ_i_* is the standard deviation of the solidified shell. The larger the uniform index is, the more uniformly solidified the shell will be. What is more, because the influence of the electromagnetic force is mainly concentrated at −0.4 m distance from the meniscus for the current stirrer position, therefore, in the current study, we use the z = −0.4 m transverse plane to analyze the uniformity. Figure 9 and Table 3 show the uniform index at the mold wide face and narrow face under different EMS currents.

As shown in Figure 9, compared with that without EMS, EMS can effectively improve the uniformity of the solidified shell, especially for the mold narrow face. For the mold narrow face, the uniform index changes little as the EMS current increases from 400 to 700 A, the main reason is that the electromagnetic driving flow can suppress the direct impact of jet flow on the mold narrow face, and benefit to the uniform growth of solidified shell. For the mold wide face, it can be observed that the uniform index increases at first and then decreases accompanied with the increase of EMS current, this can be attributed that for the high EMS current, the remelt phenomenon at region II intensified, so the uniform index decreased.

### 3.4. Effect of Casting Speeds on Fluid Flow in the Mold

Considering that the flow velocity uniformity on the left and right sides of the mold is better at 600 A, as shown in Figure 6a, and the thickness of the shell with narrow surface is thicker at 600 A, as shown in Figure 8b, the thickness of the shell with narrow surface is conducive to improving the speed. According to Figure 6a and Figure 8b, it is better at 700 A, but considering the uniform index of the solid shell as shown in Figure 9, the uniformity index of the wide face shell is worse at 700 A, therefore, 600 A was selected for further study.

**Figure 10:**
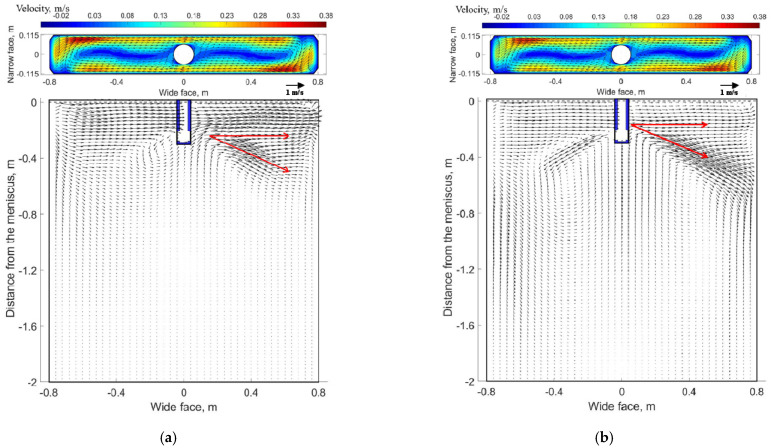
Velocity distribution at transverse plane (plane 1) and a quarter longitudinal plane (plane 2) at (600 A, 4 Hz): (**a**) 0.8 m/min, (**b**) 1.4 m/min.

The velocity distribution at plane 1 and plane 2 under different casting speeds when the EMS current is 600 A and frequency is 4 Hz are shown in Figure 10.

**Figure 11:**
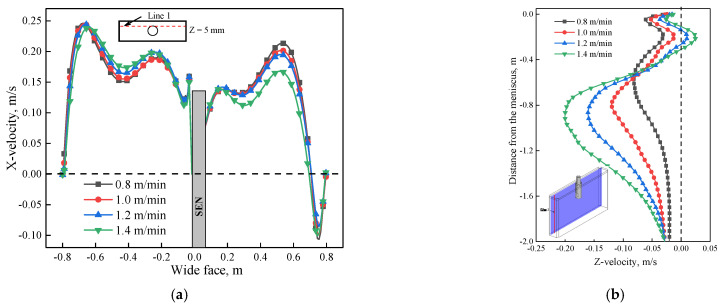
Variation of velocity under different casting speeds at (600 A, 4 Hz): (**a**) X-velocity at line 1, (**b**) Z-velocity at line 2.

As shown in Figure 10, for plane 2, molten steel tends to flow from the left side of the SEN to the right side of the SEN due to the effect of electromagnetic force. When the casting speed is 0.8 m/min, it can be seen that the jet flow at the left side of SEN has nearly disappeared by comparing it with that of 1.4 m/min. While at the right side of the SEN, the jet angle increases significantly as the casting speed increase from 0.8 to 1.4 m/min. The main reason is that under the same EMS current, for the low casting speed (0.8 m/min), the jet velocity poured from the SEN is relatively small. Therefore, under this condition, the jet flow at the left side of the SEN is constrained more significantly, while for the right side of the SEN, the jet flow can also be easily dragged under the low casting speed, and results in a small jet angle. For plane 1, although a horizonal recirculating flow has been generated for these two cases, the velocity at the decelerated region is small when the casting speed is 1.4 m/min due to the hindering effect of stronger upper recirculation flow. Figure 11a shows the variation of velocity under different casting speeds at line 1, the velocity at the left side of SEN is greater than that of the right side; because of the accelerated region, with the increase of casting speed, although a transverse flow was generated for all casting speeds, the difference of maximum velocity at both sides of the SEN increases, as casting speed increases from 0.8 to 1.4 m/min, the difference increases from 0.0188 to 0.072 m/s. For the longitudinal line (Figure 11b), for the low casting speed, a plug flow has generated near the mold wall, it means that a critical casting speed exists to avoid the plug flow.

### 3.5. Effect of Casting Speed on Solidification

**Figure 12:**
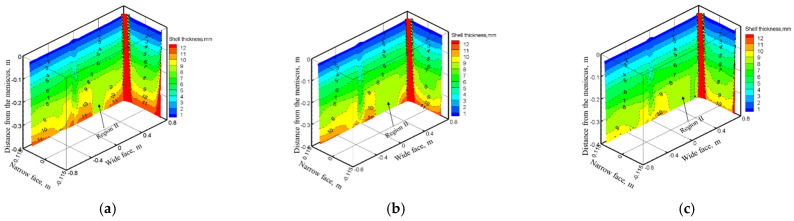
3D variation of solidified shell thickness along the casting direction under different casting speeds at (600 A, 4 Hz): (**a**) 0.8 m/min, (**b**) 1.2 m/min, (**c**) 1.4 m/min.

The variation of solidified shell thicknesses along the casting direction and center of the mold wide face and the narrow face under different casting speeds when the EMS current is 600 A are shown in Figure 12.

**Figure 13:**
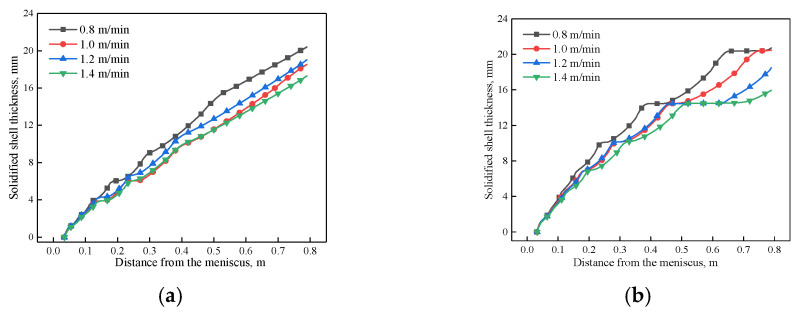
Variation of the solidified shell at the center of, (**a**) wide face, (**b**) narrow face under different casting speeds at (600 A, 4 Hz).

As shown in Figure 12 and Figure 13, the solidified shell thickness decreases with the increase of casting speed. For the mold wide face, a remelting concave has generated at region II because of the deviated jet flow induced by electromagnetic force gradient, and it is aggravated with the increase of casting speed. For the mold narrow face, it can be seen that with the increase of casting speed, the remelt phenomenon of solidified shell thickness aggravates due to the strong jet flow. When the casting speed increases from 0.8 to 1.4 m/min, the shell thickness of the wide face and narrow face at the mold exit changes from 20.72 and 20.4 mm to 15.95 and 17.31 mm, respectively.

**Figure 14:**
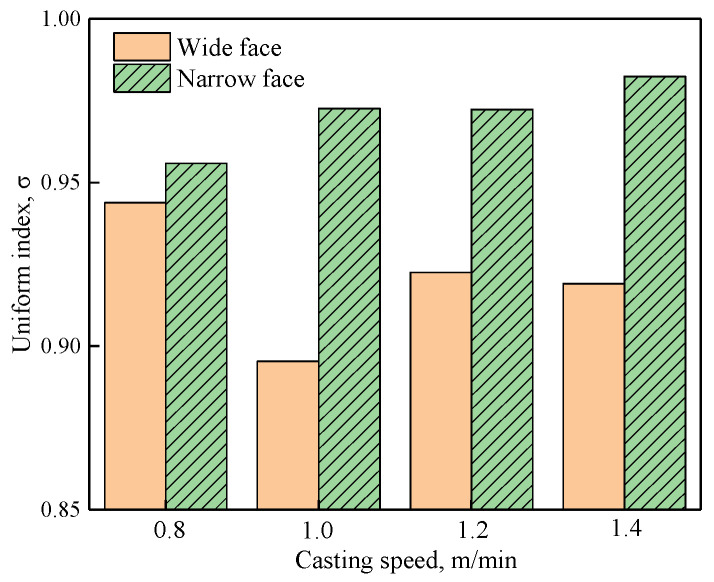
Uniform index at z = −0.4 m under different casting speeds.

The uniform index at z = −0.4 m under different casting speeds when the EMS current is 600 A is shown in Table 4. The uniform index at the mold narrow face and the wide face is shown in Figure 14. As shown in Figure 14, the uniform index at the mold narrow face is larger than that of the mold wide face, the main reason is that when EMS is applied, the jet flow which directly impacts the narrow face has been suppressed, and this may benefit uniform growth of the solidified shell, while for the mold wide face, the deviated jet flow can break the uniformity of the solidified shell. With the increase of casting speed, the uniform index at the mold wide face decreases firstly and then increases, while at the mold narrow face it increases but changes are not significant.

## 4. Application Effects

**Figure 15:**
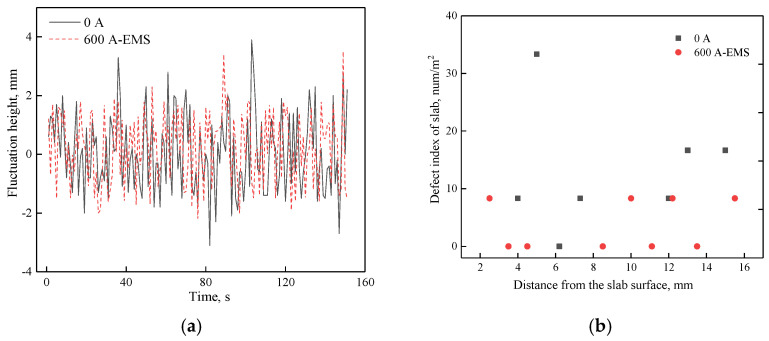
Industrial test: (**a**) transient level fluctuation at quarter of mold, (**b**) number density of inclusion.

According to the optimal parameters of the mathematical simulation, the industrial test including level fluctuation and number density of inclusion was carried out with and without EMS when the casting speed was 1.2 m/min. The relevant results are shown in Figure 15.

As shown in Figure 15a, the largest level fluctuation is less than ±5 mm whether the EMS is on or off. However, when EMS is applied, the amplitude of fluctuation level is relatively small compared to that without EMS, it indicates that EMS can decrease the fluctuation level for a suitable EMS current. In addition, the inclusion has also been detected by magnifying glass and the size which is larger than 100 μm can be observed by this method. It can be seen that the number density within 15 mm from the slab surface have a decreasing trend when the EMS is applied, which demonstrates the benefits of the optimized flow control parameters.

## 5. Conclusions

A three-dimensional mathematical model is conducted to investigate the fluid flow, solidification and level fluctuations under different EMS currents and casting speeds. The uniform index of the solidified shell thickness has been introduced to judge the uniformity of the solidified shell. Conclusions of this study are summarized as follows:

(1) When EMS is applied, a horizonal recirculating flow has been generated, accelerated and decelerated regions exist in the mold. With the increase of EMS current, the difference of velocity near free surfaces decreases; large EMS current may generate plug flow near the mold wall.
(2) EMS can obviously improve the uniformity of the solidified shell, with the increase of EMS current, the uniform index at the mold narrow face increases while at the mold wide face it first increases and then decreases.
(3) Under the same EMS current, with the increase of casting speed, the difference of velocity near the free surface increases, the uniform index at the mold narrow face changes little, while at the mold wide face it first decreases and then increases.
(4) EMS can weaken the level fluctuation and reduce the number density of inclusion for a suitable EMS current through industry test.
(5) A rational EMS current range exists to obtain optimal steel quality. In the current study, when the casting speed is 1.2 m/min, the rational EMS current is 500–600 A.

## Figures and Tables

**Table 1:** Process parameters used in mathematical simulation.

Parameters	Value	Parameters	Value
Section size of mold (mm^2^)	1600 × 230	Casting speed (m/min)	0.8, 1.0, 1.2, 1.4
Size of SEN port (mm^2^)	80 × 60	Coil number of each stirrer	36
Outer diameter of SEN (mm)	120	Turn number of each coil	20
Inner diameter of SEN (mm)	80	Stirrer center from meniscus (mm)	75
Inclination angle (°)	15	EMS frequency (Hz)	4
Submergence entry depth (mm)	170	EMS current (A)	400, 500, 600, 700

**Table 2:** Physical parameters used in mathematical simulation [28,29].

Molten steel Parameters	Value
Density (kg/m^3^) Specific heat (J/(kg·K)) Thermal conductivity (W/(m·K))	7000 720 31
Viscosity (Pa·s)	0.0065
Latent heat (J/kg)	275,000
Solidus temperature (K) Liquidus temperature (K)	1802 1812

**Table 3:** Uniform index at z = −0.4 m under different EMS currents.

EMS Current (A)	Uniform Index	
	Wide Face	Narrow Face
0	0.948	0.828
400	0.961	0.971
500	0.965	0.97
600	0.923	0.972
700	0.9	0.973

**Table 4:** Uniform index at z = −0.4 m under different casting speeds.

Casting Speed (m/min)	Uniform Index	
	Wide Face	Narrow Face
0.8	0.944	0.956
1.0	0.895	0.973
1.2	0.923	0.972
1.4	0.919	0.982

## Data Availability

Data sharing not applicable. No new data were created or analyzed in this study. Data sharing is not applicable to this article.
